# Language development in Iranian children with epilepsy

**Published:** 2020-01-05

**Authors:** Hamid Nemati, Maryam Jalalipour, Shadi Niliyeh, Behjat Maneshian

**Affiliations:** 1 Shiraz Neuroscience Research Center, Shiraz University of Medical Sciences, Shiraz, Iran; 2 Rehabilitation Sciences Research Center, Shiraz University of Medical Sciences, Shiraz, Iran; 3 Department of Speech Therapy, School of Rehabilitation Sciences, Shiraz University of Medical Sciences, Shiraz, Iran; 4 Department of Pediatrics, School of Medicine, Shiraz University of Medical Sciences, Shiraz, Iran

**Keywords:** Language Delay, Age of Onset, Children, Iran, Epilepsy

## Abstract

**Background:** Epilepsy is the most common pediatric neurologic disease accompanying with psychosocial delays causing a child’s isolation from the society. Developmental language delays are among the most common complaints of children with epilepsy. In the current study, verbal skills and expressive and receptive language development have been assessed in patients with epilepsy and compared with age-matched normal group.

**Methods:** This case-control study was conducted on 78 2-5-year-old children with epilepsy and 78 age-matched normal children referred to the outpatient clinic of Imam Reza affiliated to Shiraz University of Medical Sciences, Shiraz, Iran, in 2017-2018. Demographic information of cases (age, age of onset, type of seizure, and number of consumed remedies) and controls was gathered. In order to assess study population’s verbal, receptive, and expressive language development, Newsha growth measurement test, a validated Persian version of verbal language development questionnaire, was utilized.

**Results: **Comparison of children with epilepsy with normal controls showed a significant difference in spoken, expressive, and receptive language development between children with epilepsy and normal peers (P < 0.05). Spoken and receptive language developments were significantly in association with earlier age of onset, higher number of remedies received for seizure control, generalized type of seizures, and lacking of seizure control (P < 0.05). Expressive language development showed no association with type and control of seizures (P > 0.05) but had significant association with age of onset of epilepsy and number of remedies (P < 0.05).

**Conclusion:** Assessment of verbal language development aspects among children with epilepsy showed a higher rate of delay among these children as compared with normal age-matched ones. Moreover, earlier age of onset, generalized type of seizures, higher number of consumed remedies, and poor seizure control were accompanied with higher and more severe speech and language delay.

## Introduction

Epilepsy is the most common pediatric neurologic disease. Epidemiology of epilepsy is not well-known due to various criteria used by different scientists for definition of epilepsy, though its cumulative rate in the first five-year of life was estimated 0.45% proceeding to 0.66% within the first ten years of life.^1^ Epilepsy can lead to significant complications such as stigma, behavioral, cognitive, and psychosocial delays affecting the life of a person and his/her family in a considerably negative manner.^1^^,^^2^ Moreover, it has been estimated that up to 25% of children with epilepsy would never experience seizure control that poses more severe complications for this vulnerable population.^3^ Even those patients with well-controlled seizures may experience complications due to impaired mental function and structure, remained sequels even following epilepsy control, and adverse effects of heavy treatments used for seizure control.^4^^,^^5^

Speech and language delays are among the most common epilepsy-induced cognitive complications involving up to 15% of children with epilepsy.^6^ Various types of epilepsies have been mentioned which pose speech and language developmental delays, among them, rolandic epilepsy, temporal lobe epilepsy (TLE), absence epilepsy, and juvenile myoclonic epilepsy (JME) can be named. For instance, studies have shown that rolandic epilepsy can involve all aspects of speech and language, especially expressive language and verbal development.^7^

Early diagnosis and appropriate treatment of developmental speech and language delays among children with epilepsy can help them actively participate in communication, present successful roles in the society, and prevent their isolation. Based on our research, the number of studies assessing verbal and language developmental delay among children with epilepsy and its comparison with normal population in the community of Iran is limited. Thus, the current study has been aimed to assess verbal language development among children with epilepsy using Newsha questionnaire, a validated Persian growth measurement test.

## Materials and Methods

This case-control study was conducted on 78 2-5-year-old children with documented diagnosis of epilepsy referring to outpatient clinic of Imam Reza (affiliated to Shiraz University of Medical Sciences, Shiraz, Iran) and on 78 age-matched normal children who referred to this outpatient clinic with complaints other than pediatric epilepsy from January 2017 to March 2018.

All children with documented diagnosis of epilepsy [based on electroencephalography (EEG) and medical records, either local or generalized epilepsy) diagnosed by a target pediatric neurologist and age range of 2-5 years were included.

Exclusion criteria were:

Documentation regarding neurodevelopmental delay, cerebral palsy/mental retardation, autism, hearing impairment, and simple or complex febrile convulsion. Mentioned exclusion criteria were assessed by a target pediatric neurologist in order to prevent any interobserver bias. 

After study protocol approval by Ethics Committee of Shiraz University of Medical Sciences (code: 16211), all required information about the study was declared to cases' and controls' parents. Moreover, they were reassured about the confidentiality of their data. Thus, parents were requested to sign the written consent form of their child participation in this study.

Selection of Study population was performed using convenience sampling among all 2-5-year-old children who met inclusion criteria until achieving the goal number of 78 cases. Then, controls were age-matched with cases using convenience sampling again.

In order to include controls, the Ages and Stages Questionnaire (ASQ) was performed to confirm their appropriate development.

Demographic information of cases including age, age of epilepsy onset, status of seizure control during the recent six months, number of consumed remedies, and type of seizures (focal/generalized) was recorded. Mentioned information was gathered through patients' medical records by a target pediatric neurologist. For controls, only their age was recorded.

In order to assess study population’s spoken, receptive, and expressive language development, Newsha growth measurement test, a Persian version of verbal language development test for children from birth to six years of age, was utilized.

Newsha growth measurement test is a Persian developmental test that assesses 7 subscales including hearing, receptive language, expressive language, speech, cognition, social community, and motor development of children from their birth to six years of age. This test was validated by Jafari et al. in 2008 (Geriatric Research Center, University of Social Welfare and Rehabilitation Sciences, Tehran, Iran). General correlation of this test through test-retest experiment was over 95% for all subscales (speech: 99%, receptive language: 96%, and expressive language: 98%). Furthermore, validity and reliability of this test was assessed using Likert scale and results were as speech: 99%, receptive language: 96%, and expressive language: 98%.^8^

The questionnaires were responded by parents and then evaluated and scored. Obtained data were analyzed using SPSS software (version 20, IBM Corporation, Armonk, NY, USA). Descriptive data were presented in mean and percentages. For analysis, t-test and chi-square test were used. A P-value < 0.05 was considered as the significant level. 

## Results

This study was conducted on 78 patients with epilepsy with age range of 2-5 years as cases and 78 normal age-matched children as controls. Table 1 presents the age distribution of cases and controls as they were supposed to be evaluated based on Newsha questionnaire, an age-based language development Persian questionnaire.

**Table 1:** Age distribution of cases and controls participating in this study

**Age (month)**	**Case [n (%)]**	**Control [n (%)]**
19-24	11 (14.1)	11 (14.1)
25-30	10 (12.8)	10 (12.8)
31-36	22 (14.1)	22 (14.1)
37-42	14 (17.9)	14 (17.9)
43-48	16 (20.5)	16 (20.5)
49-60	16 (20.5)	16 (20.5)
Total	78 (100)	78 (100)

Comparison of children with epilepsy with normal controls showed that the prevalence of expressive and receptive language delay as well as spoken delay was significantly higher among cases (P < 0.05). Table 2 demonstrates the spoken and language delay among cases and controls in details.Table 3 shows the presence of verbal and language delay among cases based on age of epilepsy onset, type of epilepsy, number of remedies received for seizure control, and seizure control. A number of 56 (71.80%) children with epilepsy presented abnormal spoken development while remained 22 (28.20%) cases were normal. Expressive language development assessment showed that 20 (25.60%) cases were abnormal while, 58 (74.30%) of them were normal and evaluation of receptive language development showed that 25 (32.05%) cases presented abnormality and 53 (67.94%) cases were normal. Based on findings of this table, spoken and receptive language developmental delays were significantly in association with earlier age of onset, higher number of remedies received for seizure control, generalized type of seizures, and lacking of seizure control (P < 0.05). Assessment of expressive language developmental delay showed no association with type and control of seizures (P > 0.05), while statistical association was detected with age of epilepsy onset and number of remedies prescribed for seizure control (P < 0.05).

## Discussion

Considering our research, this is the first study in the community of Iran that has assessed spoken and language developmental delays among children with epilepsy. In the current study, speech and language development was compared between 2-5-year-old children with epilepsy and age-matched normal controls. Moreover, we included children with epilepsy whether resenting from generalized or focal seizures. 

Our findings in confirmation with previous studies presented that children with epilepsy were significantly higher affected considering spoken, receptive, and expressive language development delay than normal age-matched controls. These findings were consistent with what was presented in the literature regarding language developmental delays among children with epilepsy.^6^^,^^9^^,^^10^

**Table 2:** Comparison of cases and controls regarding verbal and language developmental delays

**Variable**		**Case group [n (%)]**	**Control group [n (%)]**	**P**
Spoken language	Abnormal	22 (28.2)	3 (3.8)	< 0.001
	Normal	56 (71.8)	75 (96.2)	
Receptive language	Abnormal	25 (32.1)	4 (5.1)	< 0.001
	Normal	58 (67.9)	74 (94.9)	
Expressive language	Abnormal	20 (25.6)	5 (6.4)	0.001
	Normal	58 (74.4)	73 (93.6)	

**Table 3:** Assessment of cases' verbal, receptive language, and expressive language developmental delays regarding age of onset, number of remedies, type, and control of seizures

**Variable**			**Delayed (n = 78)**	**Normal (n = 78)**	**P**
Spoken language	Age of onset		13.95 ± 7.53	23.05 ± 9.10	< 0.001
	Number of remedies		2.14 ± 0.88	1.32 ± 0.57	< 0.001
	Type of seizure	Focal	1 (4)	36 (64)	< 0.001
		Generalized	21 (96)	20 (36)	
	Seizure control	Controlled	9 (40)	53 (95)	< 0.001
		Uncontrolled	13 (60)	3 (5)	
Expressive language	Age of onset		15.35 ± 8.70	22.26 ± 9.27	0.005
	Number of remedies		2.00 ± 0.85	1.40 ± 0.67	0.002
	Type of seizure	Focal	7 (35)	30 (52)	0.290
		Generalized	13 (65)	28 (48)	
	Seizure control	Controlled	13 (65)	48 (85)	0.100
		Uncontrolled	7 (35)	9 (15)	
Receptive language	Age of onset		16.24 ± 8.12	22.49 ± 9.61	0.006
	Number of remedies		2.04 ± 0.79	1.32 ± 0.64	< 0.001
	Type of seizure	Focal	1 (4)	36 (67)	< 0.001
		Generalized	24 (95)	17 (33)	
	Seizure control	Controlled	16 (64)	7 (13)	0.020
		Uncontrolled	9 (36)	46 (87)	

Data are presented as mean ± standard deviation (SD) or number and percentage

Further evaluations of this study were only targeted to spoken, receptive, and expressive language development of children with epilepsy. Therefore, we found that earlier age of epilepsy onset and higher number of remedies utilized by a patient were in significant association with more severe presentations.

Similar to what was presented in the literature, we detected more severe brain sequels among those children with epilepsy with earlier age of onset; this fact may have probably occurred due to the higher rate of seizure attacks. Jurkeviciene et al. presented more severe signs of verbal language developmental delays among those with earlier age of epilepsy onset.^11^ These findings were presented by Selassie et al.^12^ and Yanli et al.^13^ who conducted their studies on rolandic-affected children, while study of Monjauze et al. again on children with rolandic epilepsy did not confirm this association.^14^

The other finding about number of remedies can be attributed to the poor control of the seizures, as successful control of seizure is directly in association with add-on therapy requirement. On the other hand, remedies’ adverse effects or cumulative adverse effects and/or combination of poor seizure control and complications induced by various remedies administration are responsible for these findings. In this order, it seems necessary to evaluate the value of add-on therapy whether it merits or not. 

Seizure control was the ratter evaluated variable of the current study that showed statistical association with verbal and receptive language developmental delay. Although no association was detected with expressive language in this regard, our hypothesis about association of number of remedies with spoken and language development due to seizure control status can be confirmed. 

Association of poor seizure control with language dysfunction may present the potential role of sequels occurred due to previous seizures in a patient’s medical history. This hypothesis was primarily raised by Monjauze et al. that poor seizure control poses verbal-language developmental delays^15^ and emphasized by further studies.^15^

Although language development assessment among children with epilepsy has been of great concern in the literature, few numbers of studies have evaluated language development aspects in details and based on our knowledge there was no study in this regard in the community of Iran. 

We have only assessed three aspects of language including spoken, expressive, and receptive entities of language development. Previous studies tried to detect language dysfunctions in greater entities as Jackson et al. examined their study considering 11 entities including verbal intelligence, verbal reasoning, lexical and semantic fluency, vocabulary, reception word recognition, object naming, spelling, verbal list learning, word reading, and delayed verbal memory.^6^

Smith et al. assessed children resenting from rolandic epilepsy as the most common type of childhood epilepsy. Although their study was observational, they reported that rolandic epilepsy involved all aspects of speech and language including phonology, writing, receptive, and expressive language.^17^ Selassie et al. conducted another study whose method was similar to ours. Assessment of entities including speech, receptive, and expressive aspects of language development showed significantly lower ability of children with epilepsy as compared to normal ones. They also presented that severity of language dysfunction was directly associated with the variety of the remedies used by a child with epilepsy.^12^ Association of variety of number of remedies received by a child with epilepsy with language developmental dysfunctions has been presented by other authors as well.^18^^,^^19^

Differences among studies may be attributed to the questionnaires used for assessing verbal language aspects or may have occurred due to studied population, as we have assessed all children with epilepsy regardless of their epilepsy type.

The last finding of this study targeted type of seizures that showed more severe status among those with generalized types considering verbal and receptive language development but not expressive aspect. Mentioned findings better clarify the importance of early language assessment and also early appropriate treatment provision for patients with epilepsy, as we have detected that patients with epilepsy regardless of their type of epilepsy are involved with language dysfunctions. Our outcomes about association of generalized seizures with worse language developmental delays are inconsistent with findings of other studies both old ones by Deonna^20^, Schoenfeld et al.,^21^ and Wheless and Butler^22^ and even recent ones by Jurkeviciene et al.^11^ and Selassie et al.^12^ Mentioned studies unanimously declared higher prevalence of language-related abnormalities among children resenting from focal seizures.

We have to confess that our study is considerably limited as we have not assessed language developmental entities thoroughly and only three aspects of language developmental delays including spoken, receptive, and expressive language entities were assessed. On the other hand, we have not utilized gold standard academic tests of language development,^23^ while our means of assessing was a validated Persian test. The other significant limitation of this study is about types of seizures that were merely divided into generalized or focal. Further evaluations with detailed type of seizures are strongly recommended as they can open noteworthy windows about type of language dysfunction in each type of epileptic syndrome. In order to generalize our findings to population of children with epilepsy, further studies with larger study population are recommended.

## Conclusion

Based on findings of this study, assessment of verbal language developmental aspects among children with epilepsy and its comparison with normal age-matched ones showed higher rate of incidence of verbal language developmental delay among children with epilepsy. Moreover, earlier age of onset, generalized type of seizures, higher number of consumed remedies, and poor seizure control were accompanied with higher rate of speech and language delays.

## Notes:

**How to cite this article:** Nemati H, Jalalipour M, Niliyeh S, Maneshian B. Language development in Iranian children with epilepsy. Curr J Neurol 2020; 19(1): 20-5.
